# Birth outcomes of singleton term breech deliveries in Jimma University Medical Center, Southwest Ethiopia

**DOI:** 10.1186/s13104-019-4442-6

**Published:** 2019-07-17

**Authors:** Fanta Assefa, Woubishet Girma, Mirkuzie Woldie, Biniam Getachew

**Affiliations:** 1 Department of Gynecology and Obstetrics, Medical Faculty, Jimma University, P. O. Box 378, Jimma, Ethiopia; 2 Department of Health Policy and Management, Faculty of Public Health, Jimma University, Jimma, Ethiopia; 3 Department of Global Health and Population, Harvard T.H Chan School of Public Health, Boston, USA; 4 Independent Public Health Consultant, Addis Ababa, Ethiopia

**Keywords:** Term breech delivery, Perinatal outcome

## Abstract

**Objective:**

Breech delivery is generally associated with higher perinatal morbidity and mortality than cephalic presentation. Hence describing the outcomes of singleton term breech deliveries in Jimma University Medical Center (JUMC), Southwest Ethiopia addresses in recommendation of improving perinatal outcomes and developing protocols in selecting eligible women.

**Results:**

The incidence of singleton term breech delivery was 5.3%. Majority, (52.8%) of them had undergone emergency cesarean delivery (C/D), and 38.9% had vaginal breech delivery. There were 14 (13.9%) intrapartum fetal deaths of whom 5.6% were recorded at JUMC. A quarter (25%) of the neonates required admission to the neonatal intensive care unit; 40.7% had perinatal asphyxia, and there were 3 early onset neonatal deaths making up a perinatal mortality rate of 157.4 per 1000 breech births. The incidence of breech delivery was relatively high. Vaginal breech delivery was lower. Significant proportions of adverse perinatal outcomes were recorded. Introduction of a protocol for managing breech deliveries to select eligible women for trial of breech delivery and strengthen training of junior health professionals regularly on how to conduct assisted vaginal breech delivery to improve perinatal outcome is recommended. Further studies to identify determinants of perinatal outcomes is recommended.

**Electronic supplementary material:**

The online version of this article (10.1186/s13104-019-4442-6) contains supplementary material, which is available to authorized users.

## Introduction

Breech presentation is defined as a fetus in a longitudinal lie with the buttocks or feet closest to the cervix. The incidence of breech presentation decreases from about 20% at 28 weeks of gestation to 3–4% at term, as most babies turn spontaneously to the cephalic presentation [1, 2]. Studies have shown that the prevalence of term breech presentation varies globally. In Malaysia the incidence was shown to be 3.8%, in India 2.1%, and in Cameroon it was found to be 2.98% [3–5]. Other studies from Nigeria and Ethiopia revealed the incidence of singleton term breech deliveries to be 2.6% and 4% respectively [6, 7].

The debate surrounding the best mode of delivery is twofold; what are the best clinical practice guidelines in terms of risk management and what are the risks versus benefits of cesarean delivery (C/D) between the fetus and the mother [8]. In the United States, C/D for breech presentation rose from 12% in 1970 to 87% in 2001. This change in clinical practice was largely due to evidence from randomized trials, particularly the Term Breech Trial, that revealed a policy of planned C/D for term breech presentation was associated with a significantly large decrease in perinatal/neonatal mortality and neonatal morbidity with only a modest increase in short-term maternal morbidity, compared with that of a policy of planned vaginal delivery [9–11].

A policy of planned C/D may not be affordable or feasible course of care for women in low-resource settings. There may be individual clinical situations in which the risks of cesarean to the mother, or her desire to avoid C/D, may outweigh the short-term risks of vaginal birth to the baby [12–16].

Breech presentation and delivery have been classified as high risk because of the increased incidence of perinatal and maternal complications. Comprehensive obstetric care and intensive neonatal care play a crucial role to decrease complications related to breech delivery [17].

Despite these globally recognized risks, not much is known about the health outcomes, and different complications of breech delivery in Ethiopia. One study [7] has been done on the impact and health outcomes of singleton breech delivery in Ethiopia but, to date, there have been no such reports from Jimma University Medical Center (JUMC). Hence, in this study we aimed to describe the maternal and perinatal outcomes of term singleton breech deliveries at JUMC, Southwest Ethiopia.

## Main text

### Methods

A prospective hospital-based cross-sectional study was conducted from July 1st to December 31st, 2014 at Jimma University Medical Center (JUMC) located in Jimma town, Southwest of Addis Ababa, Ethiopia. The study setting is a tertiary referral center for Southwest of Ethiopia.

The study population included all term pregnant women (≥ 37 weeks) admitted to the maternity and labor wards with the diagnosis of singleton breech presentation during the study period. The cases were identified as breech presentation on admission using physical examination and ultrasound by Obstetrics/Gynecology (Ob/Gyn) residents. Those women who presented with antepartum hemorrhage, uterine rupture, and fetuses with major congenital anomalies were excluded from the study.

Ten 3rd year Ob/gyn residents were recruited and trained to collect data. Labor progress and fetal heart rate using Pinnard stethoscope was followed by medical interns and midwives at admission to labor ward. All deliveries were conducted by residents and midwives assigned to labor ward. The principal investigator and co-investigator supervised and verified all data collected for consistency and accuracy and check all registries whether there are any missed cases or not on a day to day basis and there were no missed cases. A checklist for data collection was developed based on previously conducted studies [1, 3, 7]. Interview of the mothers was carried out to collect information related to background and obstetric characteristics. Review of client records, and labor ward and operation room logbooks were conducted to extract data on maternal and neonatal outcomes. Gestational age (GA) was calculated based on last normal menstrual period (LNMP) for 55 (50.9%) of the mothers. In those who don’t recall their LNMPs, early ultrasound scanning and Ballard score [18] were used. Having even a single visit to any health facility despite time of visit is considered as having antenatal care (ANC) follow up. Apgar score was used to assess condition of newborns at first, and fifth minutes after birth [19]. Employment status was categorized into employed verses unemployed, employed being those who have regular job, and paid for the job. The questionnaire was pretested for clarity before the actual data were collected. Study participants were followed from the time of admission until their discharge from the hospital. Neonatal condition was followed for 7 days post-partum. Descriptive analysis of data was carried out using SPSS (version 20.0, IBM) to generate proportions and measures of central tendency. Chi square test was done to see effect of timing of breech diagnosis either before or during labor on perinatal outcome and a P-value < 0.05 was considered to be statistically significant.

### Results

Of the total 2029 deliveries in the hospital during the study period, 108 (5.3%) were singleton term breech deliveries.

#### Characteristics of participants

The age of the study participants ranged from 16 to 40 years, with a mean age of 26.21 (± 5.13). More than half (53.7%) of the mothers were rural residents, about half (50.9%) attended primary and secondary school. Forty-seven (43.5%) of the women had their first deliveries. Majority of the women (86.1%) had ANC follow up, and in nearly half (50.9%) of them breech presentation was diagnosed during their last ANC visits. A quarter (24.6%) of them had history of previous breech delivery. Overall, 99 (91.9%) of the women had term pregnancy (Table 1).

**Table 1:** Characteristics of the pregnant women with term and post-term breech presentation, 2014, JUMC, Ethiopia (n = 108)

Variable	Frequency	Percent
Age of client (years)		
< 20	20	18.5
20–30	72	66.7
31–40	16	14.8
Mean age ± SD	26.21 (± 5.13)	
Address of the client		
Urban	50	46.3
Rural	58	53.7
Occupation		
Unemployed	81	75
Employed	27	25
Educational status		
Can’t read and write	37	34.3
Primary/secondary	55	50.9
Post-secondary	16	14.8
Parity		
1	47	43.5
2–4	46	42.6
≥ 5	15	13.9
ANC follow up		
Yes	93	86.1
No	15	13.9
Breech diagnosed		
During ANC	55	50.9
In labor	53	49.1
Previous breech delivery (N = 61)		
Yes	15	24.6
No	46	75.4
Gestational age		
Term	99	91.7
Post term	9	8.3

#### Obstetric findings

Most (88%) women were in labor on arrival to the labor ward, and 42 (38.9%) were in latent first stage of labor. Frank breech was the commonest (38%) type of presentation. Ultrasound scanning was done for three quarters of the women at admission to the labor ward. Emergency C/D was the route of delivery in 57 (52.8%), while 42 (38.9%) and 9 (8.3%) had assisted vaginal delivery and elective C/D respectively. Footling breech was the commonest indication (31%) for emergency C/D (Table 2).

**Table 2:** Obstetric findings among the study participants, 2014, JUMC, Ethiopia (n = 108)

Variable	Frequency	Percent
Stage of labor on admission		
Not in labor	13	12.0
Latent phase	42	38.9
Active phase	38	35.2
2nd stage	15	13.9
Type of breech		
Frank	41	38
Complete	36	33.3
Footling	22	20.4
Unknown	9	8.3
Ultrasound scan done at admission		
Yes	81	75
No	27	25
Mode of delivery		
Assisted vaginal breech	42	38.9
Emergency C/D	57	52.8
Elective C/D	9	8.3
Indication for emergency C/D (N = 58)		
Prolonged latent phase	10	17.2
Cord prolapse	3	5.2
NRFHR pattern	8	13.8
Big baby	5	8.6
Footling breech	18	31
Prolonged ROM	4	6.9
Arrest of cervical dilation	7	12.1
Previous C/D scar	3	5.2
Indication for elective C/D		
Previous C/D scar	5	55.6
Post term	2	22.2
Big baby	2	22.2

*C/D* cesarean delivery, *NRFHR* non-reassuring fetal heart rate, *ROM* rupture of membranes

#### Perinatal outcomes

There were 14 (13.9%) intrapartum fetal deaths, of whom 5 (4.6%) were recorded after admission to the labor ward while on follow up, and 94 (87.1%) were born alive. First minute Apgar score was between 5 and 7 for the majority (72.3%) of the neonates, and fifth minute Apgar score was > 7 for most (77.7%) of the neonates. More than six in ten (62%) of the newborns were male while 67 (62%) weighed between 2500 and 3500 g with mean weight of 2988 ± 700 g. Of the neonates born alive, twenty-seven (25%) required admission to the neonatal intensive care unit (NICU); two-fifth (40.7%) of them had diagnoses of perinatal asphyxia. Three neonates died in the first 7 days of their lives while in the NICU, making a perinatal mortality rate (PMR) of 157.4 per 1000 births among the breech deliveries (Table 3).

**Table 3:** Perinatal and maternal outcomes among the participants, 2014, JUMC, Ethiopia (N = 108)

Variable	Frequency	Percent
Fetal outcome		
Alive at birth	94	87.1
Stillbirth	9	8.3
Intra-partum fetal death	5	4.6
1st min Apgar score (N = 94)		
< 5	12	12.8
5–7	68	72.3
> 7	14	14.9
5th min Apgar score (N = 94)		
< 5	2	2.1
5–7	19	20.2
> 7	73	77.7
Sex		
Male	67	62
Female	41	38
Weight, in gram mean ± SD	2988 ± 700	
< 2500 g	22	20.4
2500–3500 g	67	62
> 3500 g	19	17.6
Admission to NICU		
Yes	27	25
No	81	75
Discharged from NICU (N = 27)		
Alive	24	88.9
Dead	3	11.1
Diagnosis at NICU (N = 27)		
PNA	11	40.7
Birth injury	6	22.2
Neonatal sepsis	10	37
Perinatal outcome after day 7		
Discharged alive	91	84.3
Stillbirth	9	8.3
Intra-partum fetal death	5	4.6
Early neonatal death	3	2.8
Maternal complications		
No complications	37	34.3
Post-partum hemorrhage	26	24
Endometritis	7	6.5
Wound infection	37	34.3
Maternal death	1	0.9

*NICU* neonatal intensive care unit, *PNA* perinatal asphyxia

Moreover, as nearly half the women (49.1%) were undiagnosed until labor, statistical analysis was done whether perinatal outcome (perinatal death, birth trauma, perinatal asphyxia, still birth, intrapartum fetal loss, 5th min APGAR score, and admission to NICU) would be affected by diagnosis of breech presentation during labor or ANC follow up and there were no statistical differences. More details can be found in Additional file 1.

#### Maternal complications

Of all the mothers, 37 (34.3%) developed wound infection, and 26 (24%) had diagnoses of post-partum hemorrhage. One maternal death was recorded in the emergency C/D group caused by post-partum hemorrhage (Table 3).

### Discussion

The incidence of term breech delivery was 5.3%, higher than previous reports, with incidences in the range of 2.4–4.7% [5–7, 20]. This high incidence in our study could be because our hospital is a tertiary hospital where many abnormal presentations are referred to or there was no practice of doing external cephalic version observed during the data collection time.

Vaginal delivery was 38.9% which was lower than other countries like 55.8% in Pakistan [20], 54.61% in Cameroon [5], 42.6% in India [4], 72.1% in eastern Nigeria [6]. In the Pakistan study they have induced those women with dysfunctional labor, whereas there was no such practice in our case which can have an effect on the lower success rate. Additionally, it can also be due to a difference in selection of cases for trial of labor in which all women had proper evaluation during their ANC follow ups, whereas in our study nearly half (49.1%) of them presented for the first time in labor without having proper evaluation. In the Cameroon study cases for trial of breech delivery were selected by obstetricians unlike ours where the women selected by trainee Ob/Gyn residents which might have an effect on lower success rate. In the Indian study they have included preterm pregnancies which can have better success rate than ours.

The PMR was 157.4 per 1000 breech births, lower than the Ethiopian study done previously [7] with a PMR of 330/1000 deliveries; this difference could be the inclusion of preterm deliveries in the previous study; the PMR is also lower than the studies done in eastern Nigeria (250/1000 deliveries), [6], and India (19.2%) [4]. In the Nigerian study 61.9% of the perinatal deaths occurred in the un-booked mothers whereas majority of the women (86.1%) in our study had ANC follow up, and in the Indian study they have included preterm pregnancies which can have effect on their higher rates of PMR. Compared to a previous study done in JUMC, where the adjusted PMR was 103/1000 deliveries [21], though influence of breech presentation was not studied, mechanical factors (55.9%) were the leading causes of all term perinatal deaths.

Fifth minute APGAR score was < 7 in 22.3% of the neonates, low APGAR score of < 7 was recorded in 17.77% of neonates delivered vaginally in the Cameroon study which is lower than our study [5], this might be because in our study most of the deliveries were attended by junior doctors and midwives with few years of experience, or most of our cases were delivered by emergency C/D. A quarter (25%) of the neonates required admission to the NICU; 40.7% of them had diagnoses of perinatal asphyxia, this may be because most of the deliveries were emergency C/Ds or may be due to the fact that this hospital is a tertiary hospital and many complicated cases are referred.

There was one maternal death in the emergency C/D group caused by PPH. Planned vaginal delivery of a term singleton breech fetus may be reasonable provided that there exists specific hospital protocol for both eligibility and labor management. In those instances, in which breech vaginal deliveries are pursued, great caution should be exercised ensuring patient informed consent and documentation of this consent [22]. During the study period, it was observed that JUMC did not have a standardized policy for the management of labor with a breech presentation.

### Conclusion

The incidence of term breech delivery was higher than earlier reports. Rate of vaginal breech delivery was lower. This was compounded with high perinatal morbidity and mortality. We recommend introduction of a protocol in managing breech deliveries to select eligible women for trial of breech delivery, and strengthen training of junior health professionals regularly on how to conduct assisted vaginal breech delivery to improve perinatal outcome. Further studies to identify determinants of perinatal outcomes in breech deliveries occurring in resource poor settings are recommended.

## Limitations

Selection bias could have occurred in our study, and determinant factors of perinatal outcomes were not studied because of small number of cases and finding of cells with 0 values in the elective C/D group. Moreover, the study was conducted in a teaching hospital that provides care to the most complicated and high-risk cases in the region. As such, the result may not be a reflective of the situation in the general population.

## Additional file

**Additional file 1: Table S1.** Perinatal outcome in relation to timing of breech diagnosis among term breech deliveries, 2014, JUMC, Ethiopia.

## Data Availability

All data supporting our findings are provided in the manuscript and its supplementary material.

## Abbreviations

ANC: antenatal care
C/D: cesarean delivery
JUMC: Jimma University Medical Center
LNMP: last normal menstrual period
NICU: Neonatal Intensive Care Unit
Ob/Gyn: Obstetrics/Gynecology
PMR: perinatal mortality rate
PPH: post-partum hemorrhage
RCOG: Royal College of Obstetricians and Gynecologists
SOGC: Society of Obstetrics and Gynecology of Canada
