# Supplementary material for: Birth outcomes of singleton term breech deliveries in Jimma University Medical Center, Southwest Ethiopia
Source: BMC Res Notes. 2019 Jul 17;12:428. doi: 10.1186/s13104-019-4442-6 (PMC6637559; doi:10.1186/s13104-019-4442-6)
Supplement: Supplementary file 1 — Additional file 1: Table S1. Perinatal outcome in relation to timing of breech diagnosis among term breech deliveries, 2014, JUMC, Ethiopia. [file 13104_2019_4442_MOESM1_ESM.docx]

Table S1: Perinatal outcome in relation to timing of breech diagnosis among term breech deliveries, 2014, JUMC, Ethiopia

| **Timing of breech diagnosis** | **Perinatal Outcome** | | | | | | | |
| --- | --- | --- | --- | --- | --- | --- | --- | --- |
|  | **Perinatal death (%)** | **Birth trauma**  **(%)** | **PNA^#^**  **(%)** | **EONS^*^**  **(%)** | **Stillbirth at admission to labor ward (%)** | **APGAR score at 5^th^ minute < 7 (%)** | **Intra-partum fetal loss**  **(%)** | **Admission to NICU^$^**  **(%)** |
| Antenatal | 4 (7.3) | 2 (16.7) | 6 (46.2) | 5 (50) | 2 (3.6) | 8 (15.1) | 2 (3.8) | 13 (25) |
| Intra-partum | 10 (18.9) | 4 (28.6) | 5 (35.7) | 5 (38.5) | 7 (13.2) | 10 (21.7) | 3 (6.5) | 14 (32.6) |
| P -value | 0.072 | 0.404 | 0.436 | 0.448 | 0.072 | 0.276 | 0.433 | 0.279 |

PNA^#^ = Perinatal asphyxia; EONS^*^ = Early onset neonatal sepsis; NICU^$^ = Neonatal intensive care unit
